# Occurrence of ESBL- and AmpC-Producing *E. coli* in French Griffon Vultures Feeding on Extensive Livestock Carcasses

**DOI:** 10.3390/antibiotics12071160

**Published:** 2023-07-07

**Authors:** Marisa Haenni, Laetitia Du Fraysseix, Pauline François, Antoine Drapeau, Tristan Bralet, Jean-Yves Madec, Thierry Boulinier, Olivier Duriez

**Affiliations:** 1 Unité Antibiorésistance et Virulence Bactériennes, ANSES—Université de Lyon, 69007 Lyon, France; laetitia.dufraysseix@anses.fr (L.D.F.); pauline.francois@anses.fr (P.F.); antoine.drapeau@anses.fr (A.D.); jean-yves.madec@anses.fr (J.-Y.M.); 2 CEFE, Montpellier University, CNRS, EPHE, IRD, 34090 Montpellier, France; tristan.bralet@cefe.cnrs.fr (T.B.); thierry.boulinier@cefe.cnrs.fr (T.B.); olivier.duriez@cefe.cnrs.fr (O.D.); 3 ANSES—Bacterial Zoonoses Unit, 94700 Maisons-Alfort, France

**Keywords:** wild birds, multi-drug resistance, ESBL, scavenging, *Gyps fulvus*

## Abstract

Despite the fact that the selective pressure of antibiotics on wild birds is supposed to be very weak, they are considered potential vectors of antimicrobial resistance (AMR). Obligate scavengers such as vultures can present high proportions of resistance to extended-spectrum cephalosporins (ESC) and multi-drug-resistant (MDR) bacteria, partially due to feeding stations that are provisioned with livestock carcasses from intensive farming. Here we investigated whether griffon vultures (*Gyps fulvus*) from two populations located in the French Alps, which feed on livestock carcasses from extensive farms, may carry such resistant bacteria. Phenotypic and genotypic characterization showed an 11.8% proportion of ESC-resistant bacteria, including five extended-spectrum beta-lactamase (ESBL)-producing and one AmpC-producing *E. coli*. The five ESBL-positive *E. coli* were clonal and all came from the same vulture population, proving their spread between animals. The ESBL phenotype was due to a *bla*_CTX-M-15_ gene located on the chromosome. Both ESBL- and AmpC-positive *E. coli* belonged to minor STs (ST212 and ST3274, respectively); interestingly, ST212 has already been identified in wild birds around the world, including vultures. These results suggest that actions are needed to mitigate the spread of MDR bacteria through wild birds, particularly in commensal species.

## 1. Introduction

Antimicrobial resistance (AMR) is a major public health issue, and resistance genes have disseminated in all environments, including the most pristine ones, mostly as a consequence of a partially uncontrolled use of antibiotics in human and veterinary medicine. While studies on the characterization of AMR genes and bacteria in humans, animals, and the food interface have been abundant for decades, the role of wildlife has been long neglected [1]. With the application of the One Health concept to AMR, most sectors involved in its transmission have been identified, and the role of wildlife has been highlighted. The burden of antimicrobial resistance is often measured by the presence of resistant *E. coli*, which is a ubiquitous bacterial species, and the WHO chose *E. coli* producing extended-spectrum beta-lactamase (ESBL) as the model target for integrated multisectoral surveillance of resistance.

Since the selective pressure of antibiotics in wildlife is supposed to be very weak, AMR in this sector is likely to reflect global anthropization and the contamination of natural environments with resistance determinants selected in non-wildlife sectors. Reports of wild birds colonized by *E. coli* resistant to extended-spectrum cephalosporins (ESC) or carbapenems (CP) and by clones circulating in humans further suggested indirect contamination through food opportunities and foraging behaviors [2]. This was, for example, documented by the identification in wild birds of the human-related pandemic clones belonging to the sequence types (ST)131 or ST38 [3,4,5], STs being defined by the commonly used multi-locus sequence typing scheme based on the sequencing of seven housekeeping genes [6]. Several studies showed that AMR epidemiology in wild birds mirrored the one in humans and/or animals in the same country, thereby supporting the hypothesis of local cross-sectorial transmissions. This was the case for Enterobacterales producing ESBLs in Sweden, France, China, and Canada [7,8,9,10] or the carbapenemases IMP-4 in Australia [11] and VIM-1 in France [12]. Consequently, given the fact that core phyla present in the avian microbiota are globally similar to those found in mammals [13], each bird species contributes in its own way to the complex pathways of antimicrobial resistance transmission, and studying each sector separately will help us understand the whole picture.

The case of vultures is of special interest, first since these birds are mostly sedentary during the breeding season, living in large colonies, and second because they are obligate scavengers, feeding on the carcasses of wildlife and livestock. Consequently, contamination of vultures by multi-drug-resistant (MDR) bacteria most likely reflects local contamination of their environment and possibly a subsequent spread of these bacteria between individuals of the same colony, notably when feeding in groups. ESBL-producing *E. coli* have been reported from Hooded vultures (*Necrosyrtes monachus*) in Gambia (54/100, 54%), from Egyptian vultures (*Neophron percnopterus*) in the Canary Islands (5/22, 22.7%), or from cinereous vultures (*Aegypius monachus*, formerly known as black vultures) in Mongolia (3/30, 10.0%) [14,15,16]. MDR and ESBL producers were also reported in European griffon vultures (*Gyps fulvus*), which are distributed from Spain to India. In Spain, several studies reporting different proportions of ESBL-producing *E. coli* isolates (from 1.1% to 30.6%) pointed out the possible acquisition of MDR bacteria from livestock carcasses (pigs or poultry). These carcasses are left in feeding stations intended to favor vultures’ survival by farmers who want to get rid of carcasses reared in intensive farms [17,18,19], i.e., farms where large numbers of animals are mostly reared indoors and where antibiotics are often used as group treatments. The importance of feeding stations in the transmission of bacteria from livestock to vultures was further exemplified by the detection of highly similar *Salmonella* Typhimurium PFGE patterns between vultures and pigs or poultry from the same region [20].

In France, griffon vultures were reintroduced in the 1990s in the Southeast region (Alps), and their population has now reached >1500 individuals. Natural recycling feeding stations were also set up [21,22], which have been mainly supplied by ovine and caprine carcasses collected from local farms where extensive farming is performed. In line with what was observed in Spain, our goal was to detect *E. coli* carried by healthy vulture individuals and to estimate the prevalence of MDR and ESBL-producing *E. coli* using phenotypic and genotypic characterizations. Given that vultures in France only feed on carcasses originating from extensive farming with limited veterinarian treatments, we expected that contamination with MDR bacteria would be reduced compared to the proportions found in Spain.

## 2. Results

### 2.1. Isolation of E. coli and Genetic Diversity and Susceptibility Testing

Over the 51 bird samples, 26 (51.0%) presented the growth of an *E. coli* isolate on non-selective plates. Among these 51 bird samples, six also presented growth on ChromID ESBL agar, suggesting ESC-resistant isolates, while none could grow on ChromID carba plates, proving the absence of carbapenem resistance. These birds originated from two sampling sites in the Alps, with 25 birds from the Baronnies and 1 from the Diois. Since multiple colony morphologies could be identified from one bird, 58 *E. coli* isolates were collected, among which 44 were kept for further analysis after de-duplication based on the phylogroup (classification of the *E. coli* population in the A, B1, B2, and D groups based on the presence/absence of three genes), MLVA type, and susceptibility profile.

**Figure 1:**
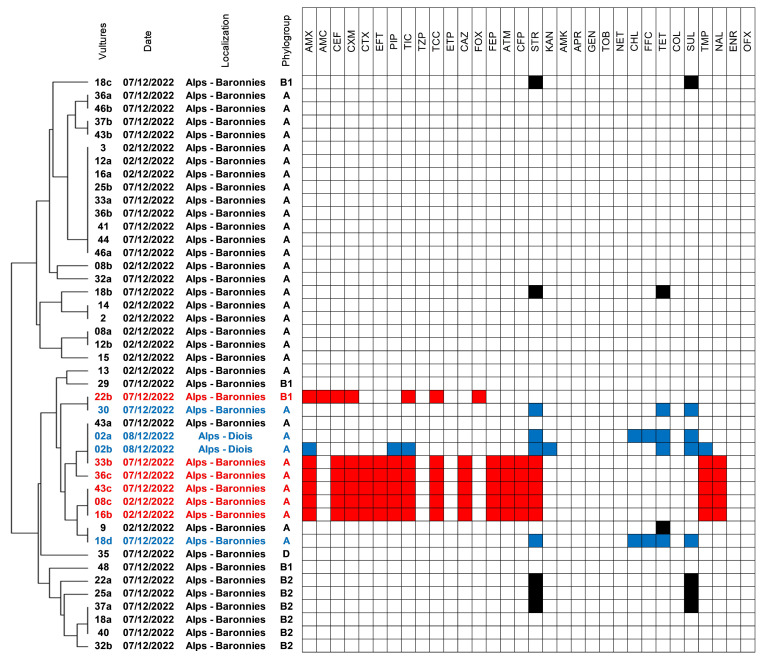
Susceptibility profiles of the 44 *E. coli* isolates collected from vultures. The genetic proximity indicated on the left part of the figure refers to MLVA results analyzed using Bionumerics. The six ESC-resistant *E. coli* were highlighted in red, while the four MDR *E. coli* were highlighted in blue.

The vast majority of the isolates belonged to the phylogroup A (*n* = 33), while phylogroups B1 (*n* = 4), B2 (*n* = 6), and D (*n* = 1) were only sporadically identified (Figure 1). No cryptic *E. coli* was found. The MLVA analysis showed that several clones circulated, some of which were identified in up to nine different birds.

### 2.2. Antimicrobial Susceptibility and Occurrence of ESBL/AmpC-Producing Isolates

Susceptibility tests showed that 28 (28/44, 63.6%) of the isolates were susceptible to all antibiotics tested.

On the contrary, six isolates (6/44, 13.6%) collected from six different birds (6/51, 11.8%) presented ESC-resistance (highlighted in red in Figure 1). These isolates were retrieved on both non-selective and selective agar; five (9.6%) presented an ESBL phenotype, and the last one (2%) presented an AmpC phenotype. The AmpC-positive isolate (strain 22b) was susceptible to all non-beta-lactam antibiotics, while the ESBL-positive ones were MDR since they presented associated resistances to streptomycin, trimethoprim, and nalidixic acid.

### 2.3. Characteristics of ESC-Resistant Isolates

The AmpC-positive isolate and two ESBL-producing isolates (considering that the five isolates presented highly similar MLVA patterns and identical susceptibility profiles) were whole-genome sequenced (Table 1).

The AmpC-positive isolate belonged to ST212; the phenotype was not due to an acquired gene but to a mutation in the AmpC promoter (Table 1). No additional resistance gene was identified.

**Figure 2:**
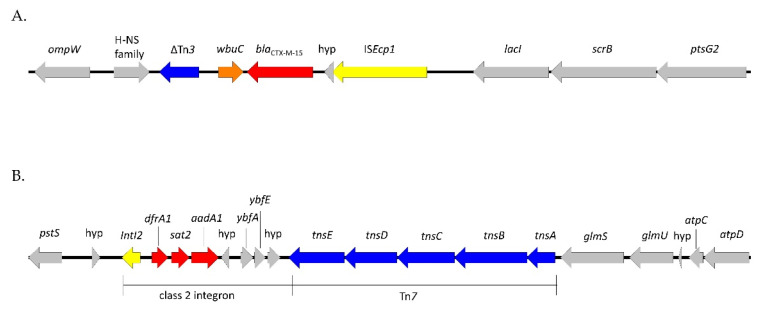
Genetic environment of the *bla*_CTX-M-15_ gene (**A**) and the Tn*7*-like element (**B**). Resistance genes are highlighted in red, the IS*Ecp1* and IntI*2* elements in yellow, the transposition modules in blue, the *wbuC* (or orf 477) gene in orange, and the chromosomal regions in gray.

The ESBL-producing isolates belonged to ST3274, and the phenotype was due to the presence of the *bla*_CTX-M-15_ gene. This gene was not plasmid-borne but inserted in the chromosome, preceded by the IS*Ecp1* element, which serves both as a promoter for the expression of ESBL and as a mobilizable transposase [23], and followed by the *wbuC* gene (also known as orf477) (Figure 2A). This genetic organization is very common for *bla*_CTX-M-15_ and has already been reported in wild birds [24]. In addition to the beta-lactam resistance, the isolates were resistant to streptomycin and trimethoprim due to the presence of the *aadA1* and *dfrA1* genes. These two genes were localized in a transposon presenting 100% homology with the MT469878 isolate [25], which is inserted on the chromosome in a different location from the IS*Ecp1*-*bla*_CTX-M-15_ element. Tn*6817* is a typical Tn*7* transposon comprising the transposition module (*tns*ABCDE), three resistance genes (*aadA1*, *sat2*, and *dfrA1*), and an inactive class 2 integrase gene integrated downstream of the *glmS* gene (Figure 2B).

### 2.4. Phylogenetic Analysis of ST212 and ST3274 Isolates

**Figure 3:**
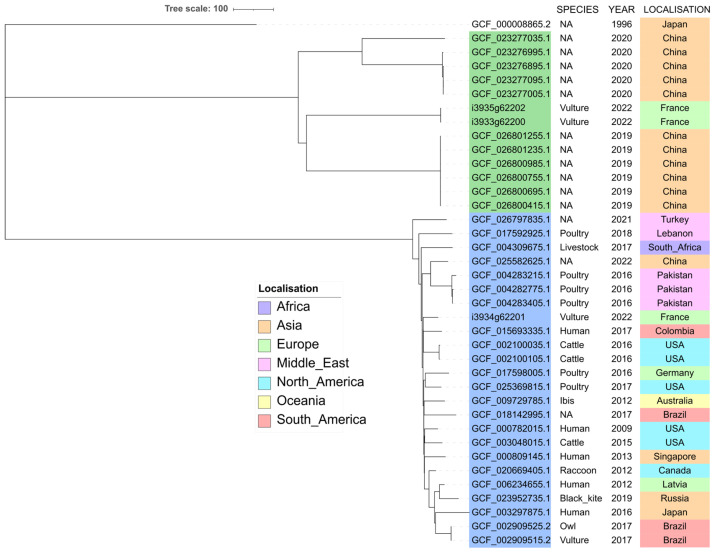
Core-genome MLST-based phylogeny of ST212 (accession numbers highlighted in light blue) and ST3274 (accession numbers highlighted in green) whole genome sequences. The tree was rooted using the outgroup *E. coli* reference genome GCF_000008865.2.

In the RefSeq database, last accessed on 11 April 2023, 32,776 *E. coli* genome assemblies were available. Twenty-three (0.07%) genomes belonged to ST212 and 11 (0.03%) to ST3274 (Figure 3), indicating that these STs are very much in the minority.

All ST3274 refer to China, except one that was isolated in Japan, but no further metadata is available.

Metadata on ST212 isolates showed that they originated from all around the world, from China to Europe, Brazil, and Canada. Ten were from farm animals (mostly poultry), six from wild animals, and five from humans. Among the six wild animals, five were collected from wild birds, including one vulture in Brazil. SNP analysis showed identity (0–2 SNP differences) between isolates from the same species and the same geographical origin, but also between two bird isolates in Brazil (a black vulture and a striped owl) with different ecological behaviors but brought to the same rehabilitation center [26]. Other isolates differed by 67–178 SNPs.

## 3. Discussion

While *E. coli* is usually systematically found in fecal samples of mammals, this is less true for birds. In this study, 51% (26/51) of the griffon vultures were *E. coli* carriers. The reported proportion of *E. coli* carriage in birds largely diverged in the literature, ranging from 36.6% in cinereous vultures from Mongolia to 86.5% in griffon vultures from Spain [18,27]. These differences might be due to bird species, geographical locations, and/or diet. However, high proportions of susceptible *E. coli* carriage in healthy birds might also reflect the degree of environmental pollution, since ESC-or CP-resistant isolates probably only represent a small part of the *E. coli* isolates contaminating vultures through scavenging behaviors.

The proportions of ESC-resistant and MDR isolates in wild birds also largely varied among studies. In Spain, ESBL-producing isolates were found in 1.1% to 30.6% of cases, while this proportion rose to 54% in Gambia [14]. In this study, the proportion of ESBL-producing isolates (5/51, 9.8%) was clearly lower than in neighboring Spain; contrary to Spain, where vultures feed mostly on intensive pig farms, vultures in the French Alps mostly feed on sheep from extensive farming, with much fewer veterinarian treatments than pigs or cows. The proportion found in France is again close to what has been found in cinereous vultures in Mongolia [16], probably due to the poorly anthropized environments of these two populations of birds [28,29,30]. The three ESBL-positive *E. coli* reported in Mongolia belonged to three different STs and presented diverse resistance genes, proving the circulation of different genetic backgrounds. On the contrary, the five isolates identified in French griffon vultures were identical; this most probably reflects the spread of a unique clone in the local population, thus evidencing the impact of close contacts between animals during feeding events on the transmission of resistant bacteria. In addition to these five ESBL-producing isolates, only four additional *E. coli* were MDR, among which were the two sole isolates collected in the Diois. Whether birds from this specific area are really more prone to carrying resistance genes remains to be documented.

WGS data showed that the ESBL phenotype was due to the presence of the *bla*_CTX-M-15_ gene, which has long been associated with the human sector before also being identified in animals and the environment. Interestingly, this gene was not carried by a plasmid but was located on the chromosome. First observed in clinical settings, chromosomal integration of *bla*_CTX-M_ genes has been increasingly reported, even in wild birds [4], and is thought to favor the stability of resistance genes in the absence of selective pressure. Indeed, maintaining a plasmid in the absence of antibiotic pressure can have an important fitness cost, but the loss of this plasmid may lead to the death of the bacteria if selective pressure reappears. Nevertheless, the *bla*_CTX-M-15_ gene, which was preceded by an IS*Ecp1* element and a hypothetical protein and followed by a tryptophan synthase beta chain and a transposase, was most probably still mobile. This genetic environment is highly similar to the one described in the IM37356 ST38 isolate collected from Mongolian wild birds [4]. Of note, the two other resistance genes identified (*dfrA1* and *aadA1*) were also localized on a mobile Tn*7* element. On the contrary, the AmpC phenotype observed in the sixth ESC-resistant isolate was due to a chromosomal mutation in the AmpC promoter and was thus not transferable.

The STs associated with ESC-resistant isolates in this study were rare STs, found in less than 0.1% of the RefSeq *E. coli* database. Consequently, it was difficult to draw hypotheses about their origin. Nevertheless, when reported, both STs were often associated with an animal, including wild birds, or an environmental source. Indeed, in addition to the isolates found in the RefSeq database, ST3274 was identified in a rook (*Corvus frugilegus)* in Poland [31], and ST212 was reported from a guinea fowl in Gambia [32], an ibis (*Threskiornis spinicollis*) in Australia [33], a primate in Senegal [34], and wastewater in Germany [35]. These different reports might indicate an environmental origin for these STs, and further studies are needed to assess whether they might be particularly present in the bird’s microbiota.

## 4. Materials and Methods

### 4.1. Ethical Approval

Vulture capture and sampling were approved by the Centre de Recherches sur la Biologie des Populations d’Oiseaux, Museum National d’Histoire Naturelle, Paris, responsible for bird ringing programs, with a delegation from the Ministry of Ecology (personal ringing programs PP345 and PP961, delivered to O. Duriez). A permit for captures and sampling was provided by the Program Personnel of the French ringing center (CRBPO, MNHN).

### 4.2. Wild Bird Sampling and Bacterial Isolation

We studied griffon vultures from the population living and nesting in Baronnies-Diois, in the French Pre-Alps. This population originated from reintroduction at two sites: in the Baronnies (44°24′ N, 5°20′ E; 56 birds released between 1996 and 2001) and in Diois (44°50′ N, 5°27′ E; 43 birds released between 1999 and 2005) [36]. This population functions as a single unit: even though twice as many birds are breeding in Baronnies compared to Diois (respectively 296 and 147 breeding pairs in 2021), most birds from both sites forage on the Diois-Vercors plateau [37]. Vultures can find food at two collective natural recycling feeding stations with vultures (NRFV), located next to the release sites and provisioned daily with sheep carcasses collected at nearby farms, and at 15 smaller “individual” NRFV spread over the region, managed by individual farmers rearing sheep. A recent GPS tracking study revealed that vultures actually find more than half of their food outside these official NRFVs, mostly carcasses of wildlife (ibex *Capra ibex*, chamois *Rupicapra rupicapra*, and red deer *Cervus elaphus*) and domestic livestock that die in the mountains [38]. Griffon vultures are colonial birds, foraging and feeding in groups, sometimes gathering several hundred individuals on a few carcasses [39].

A sample of 51 vultures was captured using permanent walk-in traps [40], baited with sheep carcasses, next to the two collective feeding stations mentioned above (2 and 7 December 2022 in Baronnies and 8 December 2022 in Diois). Birds were captured individually in the trap, brought outside, and kept on their backs on a table to collect a cloacal swab. A sample of 10 individuals were tagged with GPS-GSM loggers (as part of another study); the preliminary analysis of their tracks confirmed that they were all local birds, breeding either in Baronnies or Diois [37].

All swabs were plated on non-selective MacConckey agar and on selective media (ChromID ESBL and ChromID Carba SMART; bioMérieux) for the detection of ESC-R/CP-R Enterobacterales. One presumptive colony was picked per plate and per morphology, and *E. coli* identification was confirmed by API galleries (bioMérieux) and by species-specific PCR [41].

### 4.3. Antimicrobial Susceptibility Testing

Susceptibility testing was performed using the disc diffusion method on Mueller-Hinton agar, according to the guidelines and clinical breakpoints of the Antibiogram Committee of the French Society for Microbiology (CA-SFM; www.sfm-microbiologie.org accessed on 26 May 2023). The *E. coli* ATCC 25922 strain was used as quality control. A total of 16 β-lactam and 14 non-β-lactam antibiotics were tested. They are listed here, with the breakpoints used specified in breackets (in mm): amoxicillin (S ≥ 21; R < 14), piperacillin (S ≥ 21; R < 17), ticarcillin (S ≥ 24; R < 22), amoxicillin/clavulanic acid (S ≥ 21; R < 14), piperacillin/tazobactam (S ≥ 21; R < 17), ticarcillin/clavulanic acid (S ≥ 24; R < 22), cefalotin (S ≥ 18; R < 12), cefuroxime (S ≥ 22; R < 22), cefotaxime (S ≥ 26; R < 23), ceftiofur (S ≥ 21; R < 18), ceftazidime (S ≥ 26; R < 21), cefoxitin (S ≥ 22; R < 15), cefepime (S ≥ 24; R < 21), cefquinome (S ≥ 22; R < 19), aztreonam (S ≥ 27; R < 21), ertapenem (S ≥ 28; R < 26) tetracycline (S ≥ 19; R < 17), kanamycin (S ≥ 17; R < 15), tobramycin (S ≥ 18; R < 16), gentamicin (S ≥ 18; R < 16), amikacin (S ≥ 17; R < 15), apramycin (S ≥ 15; R < 12), netilmicin (S ≥ 21; R < 19), streptomycin (S ≥ 15; R < 13), florfenicol (S ≥ 19; R < 15), chloramphenicol (S ≥ 22; R < 19), sulfonamides (S ≥ 17; R < 12), trimethoprim (S ≥ 16; R < 12), nalidixic acid (S ≥ 20; R < 15) and enrofloxacin (S ≥ 22; R < 17). Extended-spectrum beta-lactamase (ESBL)-producing Enterobacterales were detected using the Double Disc Synergy Test (DDST).

### 4.4. Illumina Short-Read Sequencing and Data Analyses

DNA was extracted using the NucleoSpin Microbial DNA extraction kit (Macherey-Nagel, Hoerdt, France), and sequencing was performed on a NovaSeq 6000 instrument (Illumina, San Diego, CA, USA). Quality control of the reads was performed using FastQC v.0.11.9, and low-quality sequences were trimmed using Trimmomatic v.0.32. De novo assembly was performed using Shovill v.1.0.4, and the quality of assemblies was assessed using QUAST v5.0.2 (Table 1). Identification was performed using Kraken (https://ccb.jhu.edu/software/kraken/, accessed on 26 May 2023), sequence types (STs) according to Achtman’s MLST scheme, and resistance genes and virulence factors were determined using the CGE online tools (http://www.genomicepidemiology.org/, accessed on 26 May 2023). MLSTFinder v2.0, ResFinder v.4.1, and VirulenceFinder 2.0.3, while replicon content was identified using PlasmidFinder 2.0.1. Annotation was performed using the Bakta web tool (software 1.7.0, DB 5.0.0).

### 4.5. Phylogenetic Analysis

The *E. coli* A, B1, B2, or D phylogenetic groups were identified according to Doumith et al. [41]. The clonality of the isolates was estimated by multiple-locus variable-number tandem-repeat (MLVA) analysis [42]. The three isolates sequenced in the frame of this study, as well as all ST212 and ST3274 available in the RefSeq database of the NCBI database, were downloaded. The cgMLST was determined through the pyMLST pipeline v.2.0.1 (https://github.com/bvalot/pyMLST, accessed on 26 May 2023) using the scheme available on the www.cgmlst.org/ncs webpage(accessed on 26 May 2023), which comprised 2513 targets for *E. coli*. Cut-offs for highly related strains were <10 allelic variants. The resulting tree for both analyses was visualized using iTol v.6 (http://itol.embl.de/itol.cgi, accessed on 26 May 2023).

## 5. Conclusions

This study shows the occurrence of ESC-resistant isolates (11.8%) in griffon vultures in France, mostly due to the transmission through close contacts between birds of an ST212 *E. coli* clone carrying the *bla*_CTX-M-15_ gene in the chromosome. This prevalence is lower than what has been reported in Spain, probably reflecting a weaker anthropization of the birds’ environment and the fact that feeding stations were filled with carcasses of livestock (caprine, ovine) that were not reared in intensive farms. Nevertheless, surveillance of ESC-resistant bacteria should be pursued in these birds that have been successfully reintroduced in France. More globally, this study reinforces the need for large investigations clarifying the role of wildlife in the spread of AMR and its impact on environmental ecosystems and further on public health.

## Figures and Tables

**Table 1:** Characteristics and quality controls of the three fully sequenced *E. coli*.

ID	Species	ST	ESBL Resistance Genes	AmpC Mutations	Additional Resistance Genes	Plasmid Content	Genetic Support of the ESBL/AmpC Genes	Total Length	No. Contigs	N50	Coverage
33b	*E.coli*	3274	CTX-M-15	−	*aadA1*, *dfrA1*	ColpVC, IncX1	chromosome	4762230	117	107,779	377
22b	*E.coli*	212	−	AmpC-promoter (g.-42C>T)	none	Col156, IncFIB	chromosome	4900458	99	192,556	399
16b	*E.coli*	3274	CTX-M-15	−	*aadA1*, *dfrA1*	ColpVC, IncX1	chromosome	4762328	117	107,976	420

## Data Availability

The project was deposited in DDBJ/EMBL/GenBank under the BioProject accession number PRJNA976067.
